# Iatrogenic Pleural Effusion Due to Extravasation of Parenteral Nutrition via an Epicutaneo Cava Catheter in Neonates: A Prospective Cohort Study

**DOI:** 10.3389/fped.2020.570978

**Published:** 2020-10-02

**Authors:** Xinying Yu, Xuejun Wang, Ling Fan, Na Cao, Fan Yang, Jiujun Li, Hong Jiang

**Affiliations:** ^1^Department of Pediatrics, Shengjing Hospital of China Medical University, Shenyang, China; ^2^Department of Nursing, Shengjing Hospital of China Medical University, Shenyang, China; ^3^Department of Pediatrics, Plateau Medical Research Center of China Medical University, Shengjing Hospital of China Medical University, Shenyang, China

**Keywords:** neonate, Epicutaneo cava catheter, pleural effusion, parenteral nutrition, intracavitary electrocardiogram

## Abstract

**Background:** Although Epicutaneo cava catheters (ECCs) are being routinely used for intravenous access for long-term parenteral nutrition and prolonged medication administration in neonates, ECC use can be associated with rare but acute life-threatening events such as pleural effusion (PE). It is important to identify and maintain the ECC tip in a central location for preventing complications. Recently, intracavitary electrocardiogram (IC-ECG) has been developed for the real-time monitoring and verification of ECC tip position.

**Objective:** To investigate the causes and preventive measures of ECC-related PE in neonates.

**Methods:** This prospective cohort study was conducted between January 2013 and December 2017. We observed and analyzed the clinical characteristics and causes of ECC-related PE. From January to December 2017, all ECCs were guided by IC-ECG. The incidence of ECC-related PE and first-attempt success rates were analyzed before and after the introduction of IC-ECG. Additionally, the sensitivity and specificity of IC-ECG were evaluated.

**Results:** ECC-related PE was identified in 14 infants. Catheters were malpositioned in three cases; in the other 11 cases, catheters were located centrally on insertion but had migrated to non-central locations at the time of PE. After the introduction of IC-ECG, the incidence of PE was zero (*P* < 0.05). The incidence of ECC-related PE was lower when veins of the lower extremities were selected as the insertion site (*P* < 0.05). The first-attempt success rate was significantly higher in the group with IC-ECG-guided ECC placement than in the group without (*P* < 0.05). The sensitivity and specificity of IC-ECG were 97.9 and 84.6%, respectively.

**Conclusion:** ECC-related PE can be associated with either primary malposition or migration of the catheter tip. IC-ECG can help detect malposition and migration of catheter tips and improve the first-attempt success rate. Choosing a lower extremity insertion site may help decrease the rate of ECC-related PE. In neonates, IC-ECG is a reliable positioning method for ECCs with superior sensitivity and specificity.

## Introduction

Epicutaneo cava catheters (ECCs) are essential in neonatal intensive care units (NICUs), especially for the delivery of parenteral nutrition (PN). Extravasation of PN into the pleural space can cause serious pleural effusion (PE). ECC-related PE can cause acute respiratory distress and may be life-threatening without timely diagnosis and adequate interventions (1–5). Early identification and correct treatment are necessary to improve the prognosis of this devastating complication.

Previous studies have identified non-central ECC tip position as the main reason for ECC-related PE in neonates (1–6), but reports from China are rare. It is well-known that the ECC tip position is dynamic with arm movements in neonates (7). Monitoring the migration and malposition of ECCs is especially important to prevent catheter complications. Sertic et al. showed that routine radiographs were helpful for early recognition and treatment (5). Blackwood et al. recommended a low threshold for chest X-rays in neonates with even mild respiratory symptoms and transfused PN via upper extremity ECCs (2). Bashir et al. found that a large proportion of ECC-related PE occurred at ≥1 week after insertion, and they concluded that serial follow-up X-rays beginning 1 week after insertion would help to detect and identify this potentially serious adverse event (1). Given the concerns regarding excessive radiation exposure in neonates, Pezzati et al. encouraged intracavitary electrocardiogram (IC-ECG) for ECC placement monitoring (8). Recently, IC-ECG has been widely used in ECC placement. However, there are no published studies which have observed and evaluated ECC-related PE before and after the introduction of IC-ECG in neonates.

With the aim of identifying a potential strategy for decreasing ECC-related PE, IC-ECG-guided ECC tip localization was adopted at our institution. We evaluated the incidence of PE due to the extravasation of PN via ECCs in neonates before and after the implementation of IC-ECG and summarized the characteristics and causes of ECC-related PE.

## Materials and Methods

### Subjects

**Figure 1:**
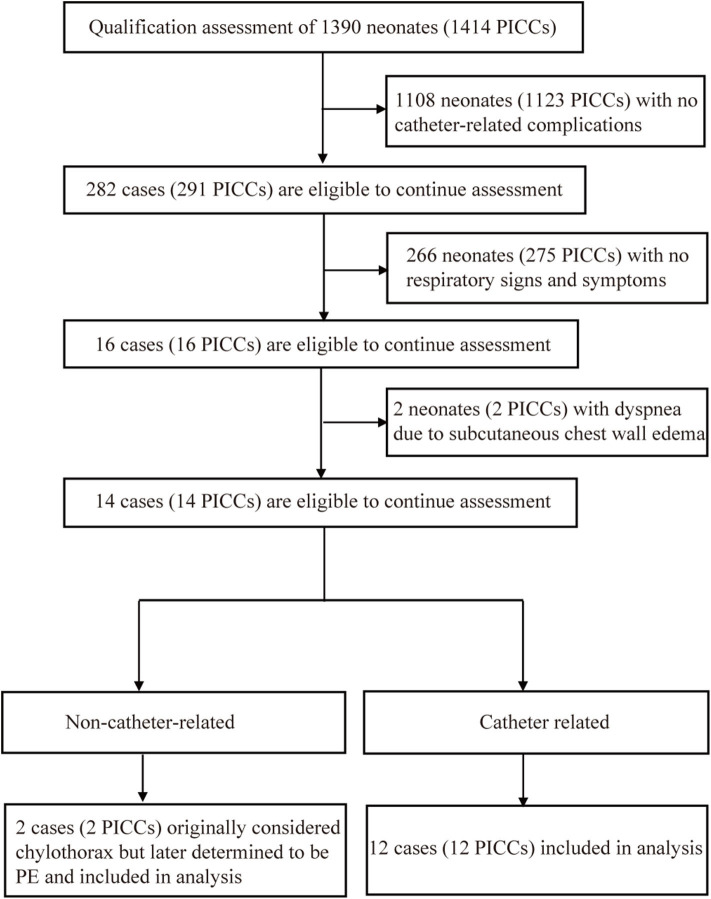
Case inclusion flowchart.

This prospective cohort study was conducted between January 2013 and December 2017 and included two observation periods, with data analysis completed in September 2019. In the first period (Non-IC-ECG-guided group), we prospectively observed and analyzed the clinical characteristics and causes of ECC-related PE in neonates who underwent treatment in the Level III NICU in Shengjing Hospital of China Medical University from January 2013 through December 2016 (Figure 1). Central positioning was defined as an upper extremity ECC tip in the superior vena cava (SVC) at T5–T7 or a lower extremity ECC tip in the inferior vena cava (IVC) at T9–T10. All catheters (UNI-ECC [1.9F, single cavity, and polyurethane]; Haolang Technology [Foshan] Co., Ltd) were inserted at bedside by certified ECC nurses, according to the ECC insertion protocol, using direct vein visualization.

In the second period (IC-ECG-guided group), we prospectively observed and summarized the incidence of PE in neonates who received ECC lines from January to December 2017. All ECCs were guided by IC-ECG using a bedside monitor COMEN C100B (Shenzhen Comen Medical Instruments Co., Ltd, Shenzhen, China). In addition, we also analyzed the first-attempt success rate before and after the introduction of IC-ECG as well as the sensitivity and specificity of IC-ECG guidance for ECC placement in neonates. The definitions for central positioning were the same as those used in the first period.

The study was approved by the Ethics Committee of Shengjing Hospital of China Medical University (approval number: 2017PS092K). IC-ECG is a non-invasive examination technique that can be performed at bedside with an electrocardiogram monitor. This study did not require patients to undergo additional tests, had no effect on the routine diagnosis and treatment of patients, and fully protected the patients' privacy. Informed consent was not required for this study according to national legislation and institutional requirements.

### ECC Placement and IC-ECG Guidelines

**Figure 2:**
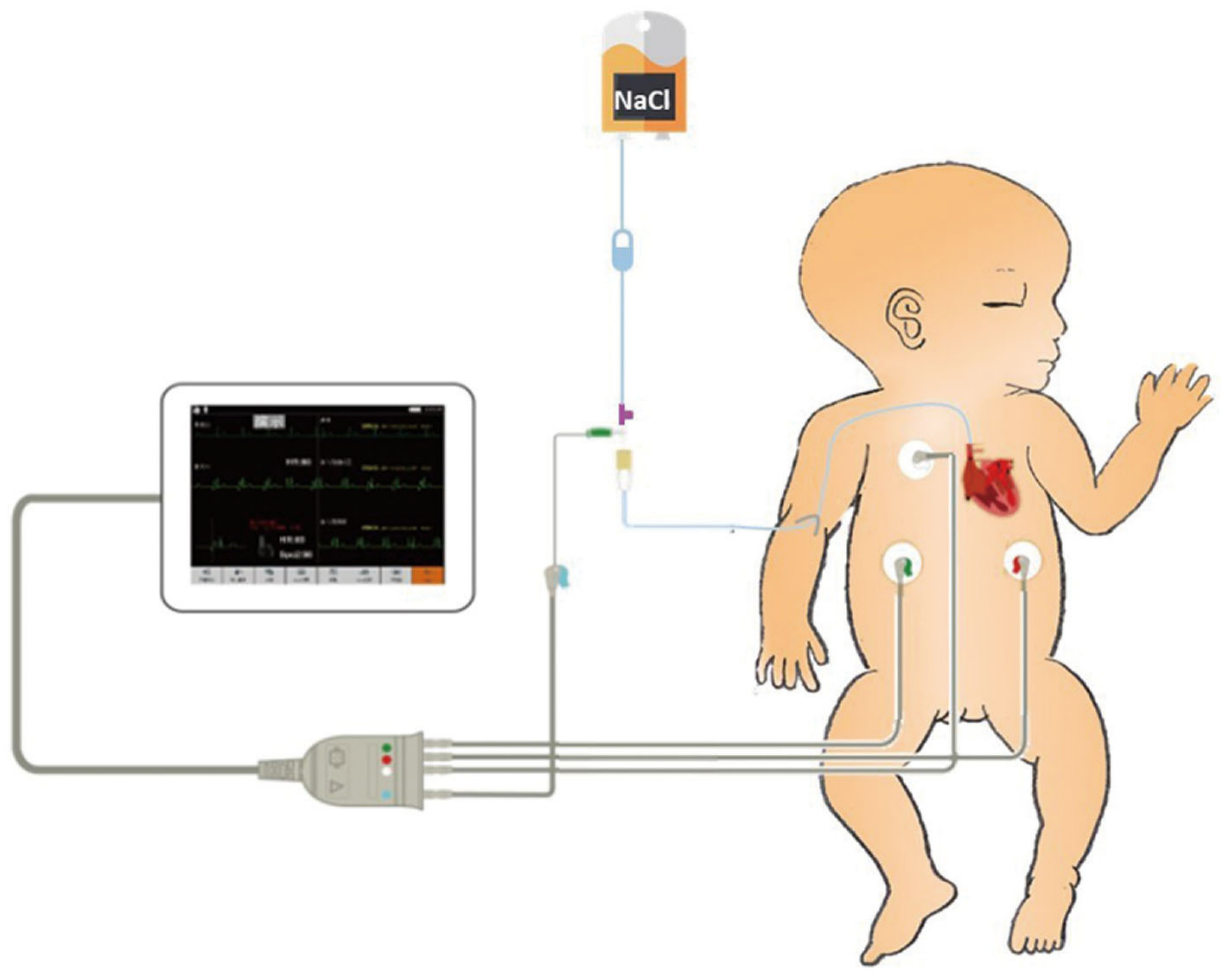
Intracavitary electrocardiogram (IC-ECG)-related products include a catheter heparin cap, scalp needle, and ECG electrode lead clip. The scalp needle (size 7) is inserted into the catheter heparin cap, leaving half of the needle outside the heparin cap. The upper left electrode is connected to the external end of the scalp needle with the ECG electrode lead clip. A column of saline in the catheter is used as an intracavitary electrode.

**Figure 3:**
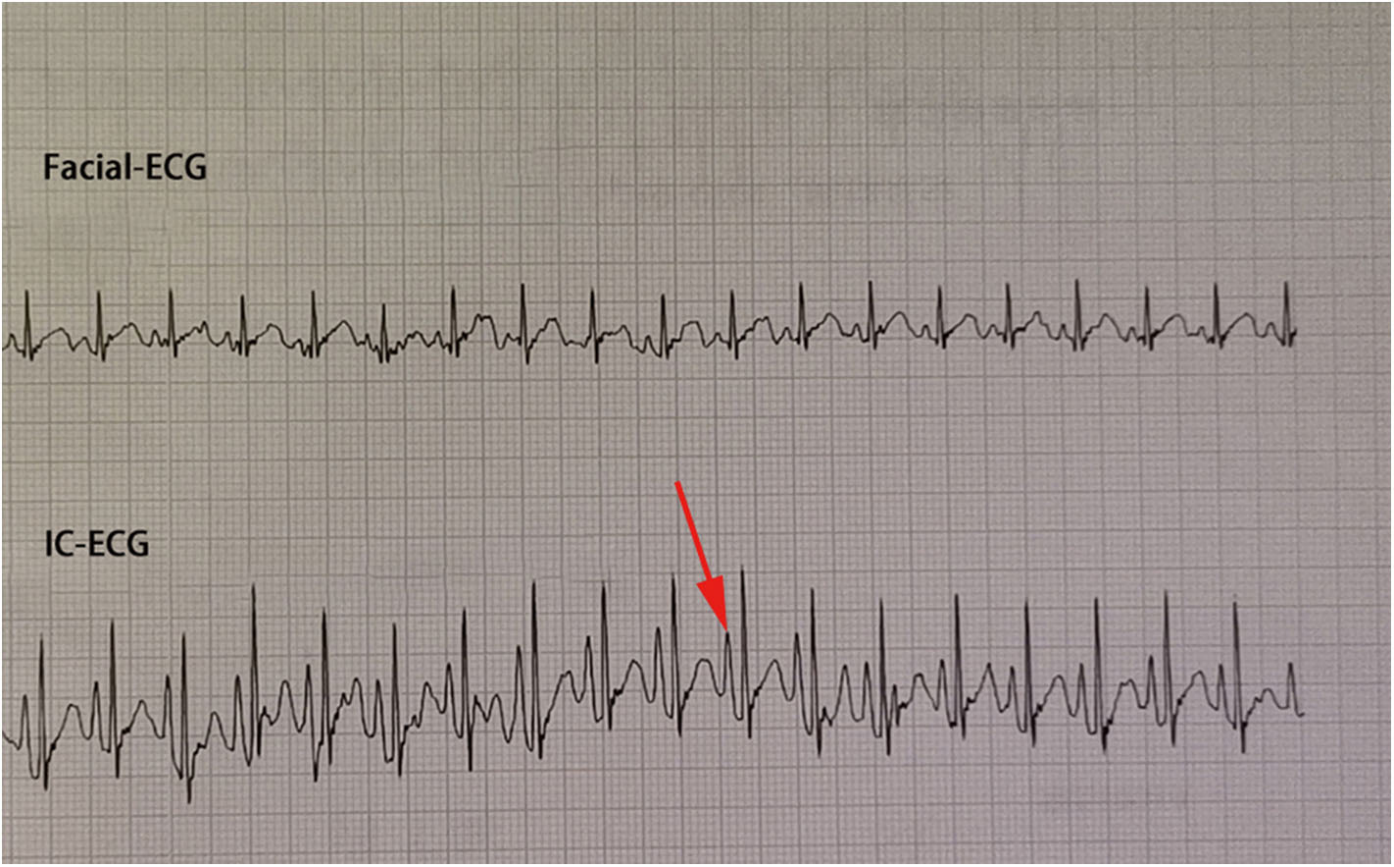
P-wave progression is seen as the catheter tip reaches the cavo-atrial junction (CAJ).

**Figure 4:**
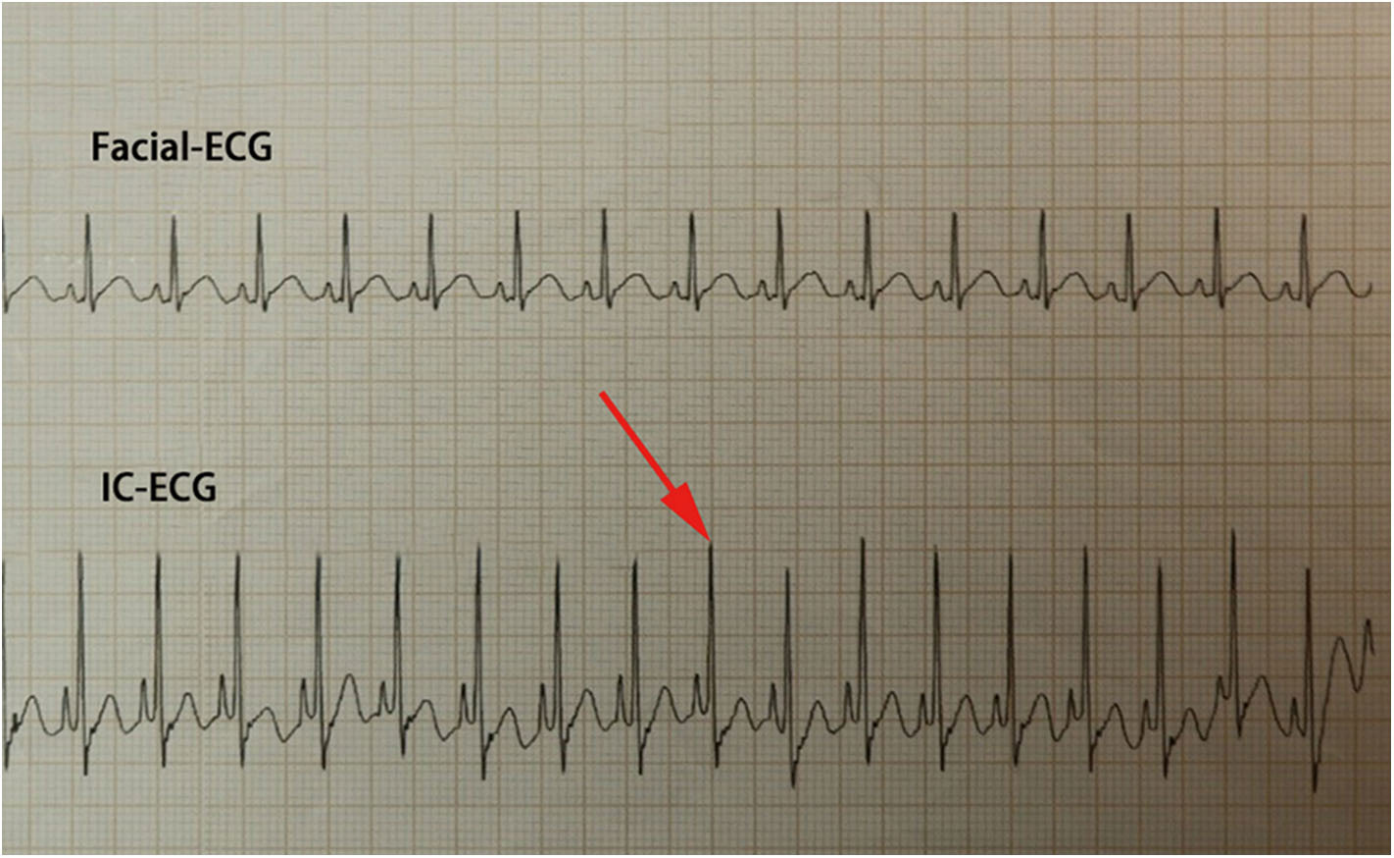
When the catheter reaches the inferior vena cava (IVC), the QRS waves continually increase in amplitude with the catheter approaching the heart. Meanwhile, the appearance of an upright P-wave indicates that the Epicutaneo cava catheter tip has reached the level of the right atrium; in this case, the catheter should be retracted.

Traditional ECC operation steps included a measurement of the catheter length based on a surface landmark, sterilization, puncture, catheterization, fixation, and postprocedural chest X-ray examination. In the IC-ECG group, prior to ECC insertion, patients were positioned in the supine position for a baseline surface lead-II ECG recording. When the catheter was advanced to the expected length, surface ECG was switched to intracavitary ECG (Figure 2) to detect dynamic changes in the P-wave with the bedside ECG monitor. The P-wave increased in magnitude as the catheter entered the SVC, reaching a maximal peak when the tip reached the correct placement at the cavo-atrial junction (CAJ; Figure 3). If the catheter was correctly placed in the IVC, the QRS waves increased in amplitude with the catheter approaching the heart (9) (Figure 4). Once correct placement was determined, the catheter was fastened, and a postprocedural chest X-ray was obtained to verify the ECC tip position as soon as possible.

In our study, most patients were preterm infants; thus, to limit X-ray exposure of neonates, in the first period of the study, we did not monitor the catheter tip locations by routine chest X-rays, but when patients developed even mild respiratory symptoms, we immediately performed bedside chest X-rays. In the second period of the study, we implemented routine weekly IC-ECG to monitor the migration of ECCs. Once a non-central position was detected by IC-ECG, a chest X-ray was needed for verification, and we adopted a proactive approach to remove the catheter or correct its malposition rather than wait for the infants to develop ECC-related PE.

### Statistical Analysis

Data analysis was performed with SPSS version 21.0 (IBM Corporation, Armonk, New York). Measurement data are expressed as means ± standard deviations, whereas count data are described as percentages. Between-group comparisons were completed using two-sided *t*-tests and χ2 tests for quantitative and qualitative variables, respectively. A *P* < 0.05 was considered statistically significant.

## Results

During the first study period, 1,390 neonates received 1,414 ECCs, 395 of which were inserted via the lower extremities, accounting for a total of 23,203 catheter days. ECC-related PE was identified in 14 neonates (Table 1), with an incidence of 0.6 per 1,000 catheter days (1.0% of ECCs). PE samples from 10 neonates were sent for laboratory testing (Table 2). Catheter tips were in primary malposition in three cases. In the remaining 11 cases, catheter tips were centrally located at the time of insertion but had migrated to non-central positions at the time of PE detection. All 14 patients eventually recovered and were discharged. The first-attempt success rate was 85.0%.

**Table 1:** Characteristics of neonates in the NICU who developed ECC-related PE.

**Characteristics**	**Patient**													
	**A**	**B**	**C**	**D**	**E**	**F**	**G**	**H**	**I**	**J**	**K**	**L**	**M**	**N**
Sex	Male	Male	Male	Male	Female	Male	Male	Male	Female	Male	Male	Male	Male	Male
Delivery Mode	CS	ND	ND	ND	CS	CS	ND	CS	CS	ND	ND	CS	ND	CS
GA, weeks	33+6/7	25+6/7	36+2/7	28	30+5/7	29+5/7	29+6/7	31+1/7	32	28+6/7	28+6/7	27+3/7	27	28
BW, g	1,620	818	2,700	1,098	1,560	963	1,211	1,333	1,405	987	1,166	900	1,143	1,189
Age at ECC insertion, days	2	3	2	2	10	3	1	2	1	2	2	5	2	4
Site of insertion	Upper limb	Upper limb	Scalp	Upper limb	EJV	Upper limb	Scalp	Upper limb	Upper limb	Upper limb	Upper limb	Upper limb	Upper limb	Lower limb
Location of the ECC tip at PE	T3	T2–T3	T1	T4	T10	T1	T4	T2–T3	T3–T4	T2–T3	T3	T1–T2	T2–T3	T10
Interval between ECC insertion and PE, days	3	37	11	8	1	16	28	5	4	14	8	2	18	7
Presenting symptoms and therapy	Apnea, ARD, NV	Apnea, ARD, NV	IVS	Apnea IOR, NV	Apnea, IOR	Apnea, ARD, NV	Apnea, ARD, NV	Apnea, ARD, NV	IOR, ARD, NV	Apnea, ARD, NV	Apnea, ARD, NV	IVS	Tachypnea ARD, NV	IVS
PE interventions	TOP, CTI	TOP, CTI	TOP, T	TOP, T	Withdraw ECC 2 cm	TOP, T	TOP, T	TOP, T	TOP, T	TOP, T	TOP, T	Withdraw ECC 2.5 cm, T	TOP, T	TOP
Days to effusion disappearance	9	10	3	1	1	1	3	1	1	1	1	1	2	1

*ARD, acute respiratory distress; BW, birth weight; CS, cesarean section; CTI, chest tube insertion; ECC, Epicutaneo cava catheters; EJV, external jugular vein; GA, gestational age; IVS, increasing ventilator settings; IOR, increased oxygen requirement; ND, natural delivery; NV, needing ventilation; PE, pleural effusion; TOP, taking out ECC; T, thoracentesis*.

**Table 2:** Details of pleural effusion in patients who underwent laboratory testing.

**Pleural effusion**	**Patient**									
	**A**	**B**	**C**	**D**	**F**	**G**	**H**	**I**	**K**	**L**
Location	Bilateral	Bilateral	Bilateral	Bilateral	Right	Right	Bilateral	Bilateral	Bilateral	Right
Depth, cm	Right: 3.5; Left: 2.5	Right: 3.2; Left: 0.7	Right: 3.1; Left: 3.2	Right: 2.6; Left: 0.8	2.7	3.6	Right, 2.1; Left, 0.6	Right, 3.2; Left, 1.7	Right, 2.3; Left, 2.4	1.8
Color	Milky yellow	Milky white	Milky white	Milky white	Milky yellow	Milky white	Milky white	Milky yellow	Milky white	Milky white
Total protein, g/L	28.1	49.0	126.4	54.7	28.9	37.9	48.9	46.7	-	29.1
Glucose, mmol/L	49.1	38.6	7.1	13.9	29.3	43.3	4.6	7.0	-	25.4
White blood cell count, /μL	50	374	100	65	8	175	157	204	175	104
Lymphocyte percentage, %	30	22	54	66	28	53	36	53	5	40

In the second period of the study, IC-ECG was routinely used for ECC guidance in all 595 neonates. Demographic characteristics between IC-ECG-guided group and Non-IC-ECG-guided group were not statistically significant (*P* > 0.05; Table 3). The incidence of ECC-related PE was zero (*P* < 0.05). The first-attempt success rate was 97.8%, significantly higher than that in the first period (*P* < 0.05). The incidence of ECC-related PE was lower when veins of the lower extremities were selected as the insertion site (*P* < 0.05; Table 4). When chest X-ray was used as the standard method for determining ECC tip location, the sensitivity and specificity of IC-ECG were 97.9 and 84.6%, respectively, with a false negative rate of 2.1% and a false positive rate of 15.4%. The crude agreement rate was 97.7% (Table 5).

**Table 3:** Comparison of baseline demographics between the two groups.

	**IC-ECG-guided** ***n*** **= 595**	**Non-IC-ECG-guided** ***N*** **= 1,390**	**t/X^**2**^**	***P***
Sex, *n* (%)			3.299	0.069
Male	254 (42.7)	735 (52.9)		
Female	341 (57.3)	655 (47.1)		
Gestational age, weeks	30.94 ± 2.71	30.97 ± 2.80	0.285	0.776
Birth weight, g	1358.81 ± 581.76	1375.42 ± 632.38	0.549	0.583
Primary diagnosis on admission, *n* (%)			3.297	0.192
Prematurity <37 weeks, *n* (%)	539 (90.6)	1220 (87.8)		
Medical	18 (3.0)	53 (3.8)		
Surgical	38 (6.4)	117 (8.4)		

*IC-ECG, intracavitary electrocardiogram*.

**Table 4:** Comparison of the insertion site, first-attempt success rate, and rate of PE between the groups.

	**IC-ECG-guided *n* = 595**	**Non-IC-ECG-guided *n* = 1,414**	**t/X^**2**^**	***P***
Site: lower limb, *n* (%)	346 (58.2)	395 (27.9)	164.245	0.000
First-attempt success rate, *n* (%)	582 (97.8)	1202 (85.0)	69.077	0.000
Rate of PE, *n* (%)	0 (0)	14 (1.0)	5.932	0.015
Age at ECC insertion, days	6.56 ± 12.45	6.04 ± 11.59	−0.898	0.369
ECC dwell time, days	16.73 ± 6.50	16.41 ± 6.88	−0.973	0.331

*ECC, Epicutaneo cava catheters; IC-ECG, intracavitary electrocardiogram; PE, pleural effusion*.

**Table 5:** The sensitivity and specificity of intracavitary electrocardiogram.

**IC-ECG**	**Chest X-ray**		**Sum**
	**+ *n* (%)**	**– *n* (%)**	
+	570 (95.8)	2 (0.3)	572 (96.1)
–	12 (2.0)	11 (1.9)	23 (3.9)
Sum	582 (97.8)	13 (2.2)	595 (100)

(+) indicates the detection of correct positioning and (–) indicates the detection of incorrect positioning.

*IC-ECG, intracavitary electrocardiogram*.

## Discussion

As ECCs are more stable than short peripheral cannulae, they are increasingly being used in NICUs in China to reduce the risk of extravasation injury from hyperosmolar PN solutions and medications. However, they can occasionally cause deadly extravasations, such as PE (1, 5, 10). Placing the catheter tips as close to the right atrium as possible and ensuring that they remain there are very important, because the wall of the vena cava is much thicker there and will be less likely to erode, in addition, this position provides increased volume and turbulence to help dilute the hyperosmolar fluid, which seems to be a factor in ECC-related PE (2). Failure to achieve or maintain optimal catheter tip placement may result in PE, which can result in death if it is associated with sudden clinical deterioration or if the cause of PE cannot be rapidly identified. In our cases, ECC-related PE was originally misdiagnosed as chylothorax in two patients (A, B). Both patients had recurrent PE due to PN infusion via ECCs, and chest tube insertions were needed until the ECCs were removed. The pleura is one of the most common sites of PN extravasation (1). The presence of high levels of glucose and low cell counts in the drained fluid suggests ECC-related PE (11, 12). An accurate diagnosis is the cornerstone of effective treatment planning and therapy.

**Figure 5:**
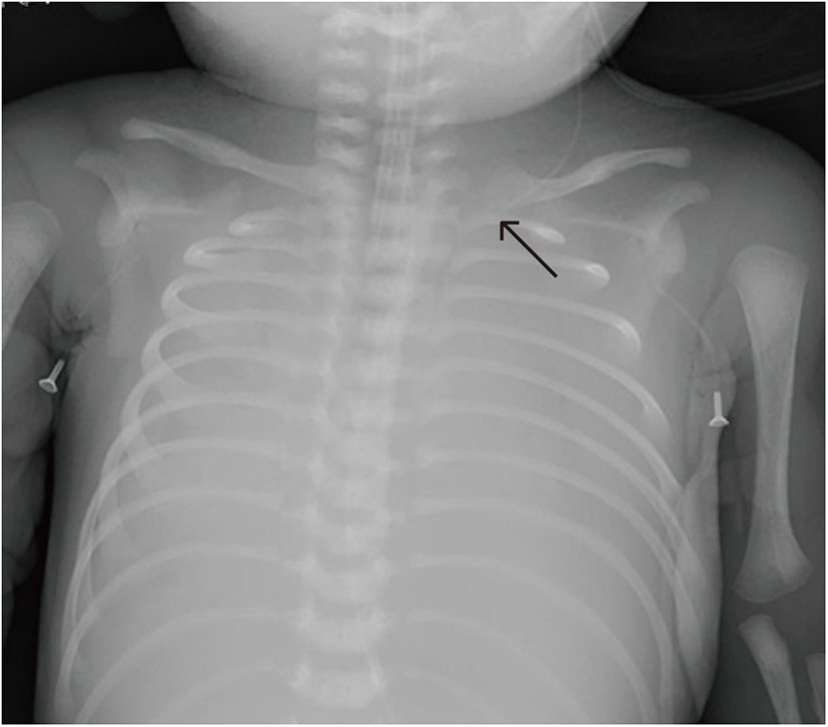
Patient C was transferred from a local hospital because of acute respiratory distress. Continuous parenteral nutrition infusion via a non-central Epicutaneo cava catheter (ECC) resulted in PE, with the chest X-ray showing bilateral “white lung”.

**Figure 6:**
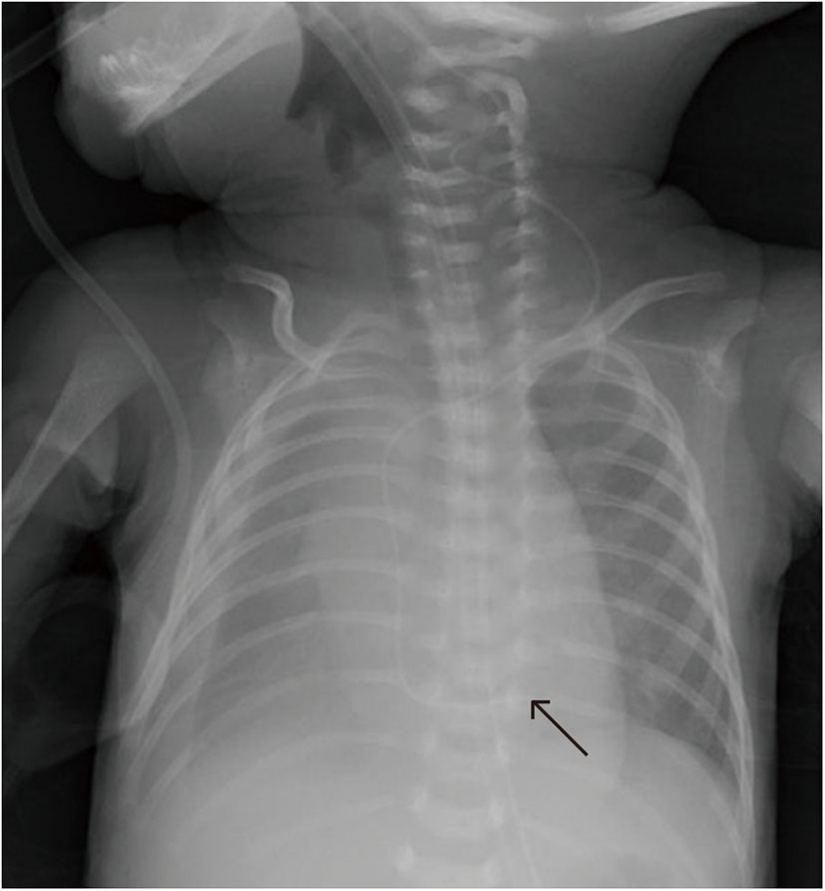
Patient E underwent uneventful Epicutaneo cava catheter (ECC) insertion, but several hours after ECC infusion, the routine postoperative chest X-ray showed that the tip was too deep.

**Figure 7:**
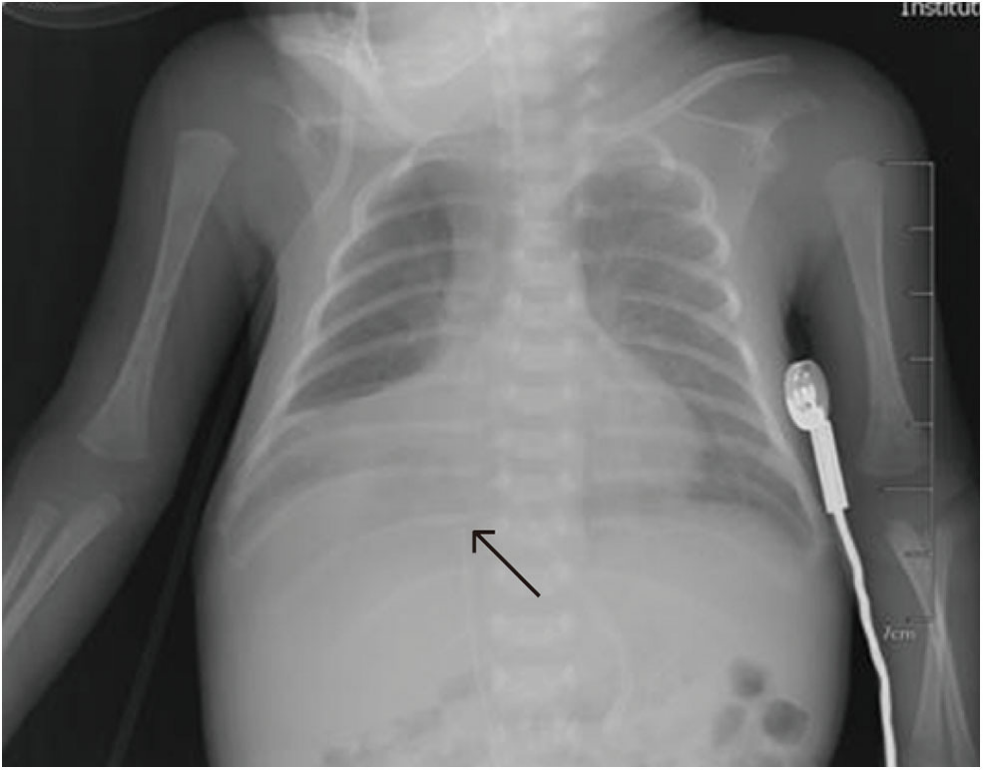
In patient N, the catheter was inserted in the great saphenous vein, and chest X-ray shows that it had reflexed. After correction, it was located at T10, and X-ray revealed pleural effusion (PE) 7 days later.

To avoid catheter-related complications and achieve maximum benefits from ECCs, the catheter tip should be centrally located (6, 13). In our study, locations of an upper extremity ECC tip in the SVC at T5–T7 and a lower extremity ECC tip in the IVC at T9–T10 were defined as the central positions. Based on the findings of our serial cases and published reports, the root cause of ECC-related PE was non-central position of the catheter tip, including a primary or secondary malposition (tip migration). Only three cases (patients C, E, and N) involved primary malposition (Figures 5–7). In the remaining 11 cases, the catheters were centrally located at insertion, but at the time of PE, they had migrated to the subclavian vein (patients B, F, H, J, L, and M), brachiocephalic vein (patients A, G, I, and K), or upper SVC (patient D). More than half of the migrations (6/11) were to the subclavian vein. Jain et al. suggested that the use of ECCs with the tip in the subclavian vein should be discouraged in neonates because of the higher rate of infiltration (14). It is important to limit ECC movement after placement and to maintain its central tip position.

In reports of ECC-related PE, most catheters were inserted from an upper extremity vein (1–4, 6, 13, 15) but few were inserted from a lower extremity vein. Kua et al. reported a case of ECC-related PE, in which the catheter was inserted from a lower extremity vein and located in the right iliac vein before migration to one of the tributaries of the azygous vein (12). The azygous vein is a low-flow system, that is at an increased risk of erosion, especially when hyperosmolar fluid is administered, such as in PN. This leads to hyperosmotic endothelial damage and increased vascular permeability, which may result in ECC-related PE (15). Based on this, we speculated that patient N's catheter had initially been located in one of the tributaries of the azygous vein rather than in the IVC, which was indistinguishable on plain X-ray. In summary, a non-central tip position was more likely to result in complications and non-selective ECC removal (6, 13).

Limb movement, neck position, forceful flushing, and neonatal growth during insertion and the dwell period may change the tip position of a peripherally inserted central catheter (PICC) line (3, 5). Despite best efforts, catheter tips may not start or stay in a central location. It is crucial to monitor the position of the ECC tip with a safe, accurate, easy, and inexpensive method, such as IC-ECG or echocardiography, to avoid malpositioning. In this study, most patients were preterm infants; thus, serial chest X-rays were not recommended to limit the X-ray exposure of neonates (8). Recently, IC-ECG-guided tip positioning has been widely used for real-time monitoring and verification of ECC tip placement via the upper extremities (9, 16–26), but there are only a few published studies on IC-ECG with ECCs inserted from the lower extremities in neonates (9). The latest multicenter study and meta-analysis both showed that IC-ECG had a more favorable positioning accuracy than the traditional X-ray method for PICC placement in adult patients (27, 28). Weber et al. also reported that IC-ECG could be used to confirm proper PICC tip repositioning, thereby reducing the need for serial radiographs (29). Repeated post-procedural monitoring of tip position by radiology is unsafe and expensive, while repeated post-procedural monitoring of tip position by IC-ECG is safe but less accurate than echocardiography. Some previous studies have regarded real-time ultrasound as the most accurate method (30, 31), but ultrasound equipment is not as widely used as bedside ECG monitor; furthermore, there is no bedside echocardiogram available in our NICU. Additionally, the use of ultrasound for tip location requires training. IC-ECG is easier in terms of operation and interpretation as it can usually be performed by a certified ECC nurse alone; thus, it is more commonly used in clinical settings in China.

After the application of IC-ECG-guided ECC tip localization, the incidence of PE decreased from 1.0 to 0% (*P* < 0.05). The first-attempt success rates before and after IC-ECG were 85.0 and 97.8%, respectively (*P* < 0.05). IC-ECG can help to improve the first-attempt success rate, avoid repeated adjustment, and save time. IC-ECG had a high sensitivity and specificity (97.9 and 84.6%, respectively), and was helpful in clinical practice. Overall, IC-ECG is a safe, accurate, efficient, cost-effective, easy to use, and replicable way of correctly confirming ECC tip position (19, 20, 25, 26). There was a significant difference in the catheterization site before and after implementation of IC-ECG (*P* < 0.05). After the implementation of IC-ECG, lower extremity insertion sites were more frequently chosen, which may have contributed to the decreased PE rate (1, 5, 6, 12). This shift occurred due to changes in cognition and experience. In the initial training, staff were advised that the upper extremity veins were preferred for ECC insertion while the lower extremity veins should be avoided as much as possible. However, in long-term clinical practice, certified ECC nurses gradually found that ECC placement was smoother when done through the lower extremity veins, with a lower incidence of ECC-related PE.

In this study, all catheters were made of polyurethane. Yu et al. reported that polyurethane catheters were stiffer and less flexible, and thus could more easily damage the vascular wall when placed in the upper extremity veins due to the curve of the aortic arch; therefore, they recommended choosing lower extremity veins (e.g., the saphenous approach) to avoid the aortic curve (12). Srinivasan et al. reported that upper limb ECCs had the highest rate of migration 24 h post-insertion compared to lower limb ECCs which showed no migration (32). When feasible, the use of a lower extremity site could help avoid ECC-related PE (2). The IVC is straighter, less branched, and has fewer influencing variables, and the accuracy of IC-ECG guidance is higher in the lower extremities than in the upper extremities (26). However, more large-scale, prospective, randomized studies are required to evaluate the incidence of PE in neonates with upper vs. lower extremity insertion sites.

## Limitation

There are some limitations in our study. Firstly, we used radiology as the standard method for assessing the ECC tip position. It would have been more appropriate to compare IC-ECG to echocardiography. Secondly, the choice of ECC site was not random, especially in the second period of the study, when lower limbs were more frequently chosen as the insertion sites. Thirdly, this was not a concurrent controlled trial but a longer historical controlled study, and the evaluation periods were not equal (4 years vs. 1 year). Finally, this study was a single center study with a relatively small sample size, and further multicenter and large-scale studies are needed.

## Conclusion

High-osmolarity PN administered via ECC can cause PE. ECC-related PE is a rare but devastating complication that can occur at any time after insertion due to primary malpositioning or migration of a central catheter tip to a non-central location; furthermore, ECC-related PE should be differentiated from chylothorax. It is crucial to monitor ECC tip position in the dwell period. Based on our case series, IC-ECG-guided ECC tip localization should be considered to detect the malposition and migration of catheter tips. In addition, choosing lower limb veins as the insertion site may help decrease the incidence of ECC-related PE.

## Data Availability Statement

All datasets presented in this study are included in the article/supplementary material.

## Ethics Statement

The studies involving human participants were reviewed and approved by Ethics Committee of Shengjing Hospital of China Medical University. Written informed consent for participation was not provided by the participants' legal guardians/next of kin because: IC-ECG is a non-invasive examination technique that can be performed at the bedside with an electrocardiogram monitor. The study did not require patients to undergo additional tests, had no effect on routine diagnosis and treatment of patients, and fully protected patients' privacy. Informed consent was not required for this study according to national legislation and institutional requirements.

## Author Contributions

XY and HJ were responsible for study conception and the design of the project. XY obtained funding and drafted the manuscript. XW, LF, NC, and FY performed acquisition of data, statistical analysis, and interpretation of data. HJ supervised the project. JL revised the manuscript. All authors reviewed and approved the final manuscript.

## Conflict of Interest

The authors declare that the research was conducted in the absence of any commercial or financial relationships that could be construed as a potential conflict of interest.

## References

[1] Bashir RA Callejas AM Osiovich HC Ting JY. Percutaneously inserted central catheter-related pleural effusion in a level III neonatal intensive care unit: a 5-year review (2008-2012). J Parenter Enteral Nutr. (2017) 41:1234–9. 10.1177/0148607116644714 27084698

[2] Blackwood BP Farrow KN Kim S Hunter CJ. Peripherally inserted central catheters complicated by vascular erosion in neonates. J Parenter Enteral Nutr. (2016) 40:8905. 10.1177/0148607115574000 25700180

[3] Kumar JKC Mukhopadhyay K Ray S. A misplaced peripherally inserted central catheter presenting as contralateral pleural effusion. BMJ Case Rep. (2018) 2018:bcr2018224471. 10.1136/bcr-2018-224471 29666098 PMC5905790

[4] Sancak S Tuten A Yildirim TG Karatekin G. Massive pleural effusion on the contralateral side of a venous peripherally inserted central catheter. J Clin Ultrasound. (2018) 46:140–4. 10.1002/jcu.22493 28440869

[5] Sertic AJ Connolly BL Temple MJ Parra DA Amaral JG Lee KS. Perforations associated with peripherally inserted central catheters in a neonatal population. Pediatr Radiol. (2018) 48:109–19. 10.1007/s00247-017-3983-x 28986615

[6] Wrightson DD. Peripherally inserted central catheter complications in neonates with upper versus lower extremity insertion sites. Adv Neonatal Care. (2013) 13:198–204. 10.1097/ANC.0b013e31827e1d01 23722492

[7] Nadroo AM Glass RB Lin J Green RS Holzman IR. Changes in upper extremity position cause migration of peripherally inserted central catheters in neonates. Pediatrics. (2002) 110:131–6. 10.1542/peds.110.1.131 12093958

[8] Pezzati M Filippi L Chiti G Dani C Rossi S Bertini G. Central venous catheters and cardiac tamponadein preterm infants. Intensive Care Med. (2004) 30:2253–6. 10.1007/s00134-004-2472-5 15517163

[9] Zhou LJ Xua HZ Xu MF Hu Y Lou XF. An accuracy study of the intracavitary electrocardiogram (IC-ECG) guided peripherally inserted central catheter tip placement among neonates. Open Med. (2017) 12:125–30. 10.1515/med-2017-0019 28730171 PMC5471914

[10] Currarino G Migration of jugular or subclavian venous catheters into inferior tributaries of the brachiocephalic veins or into the azygos vein, with possible complications. Pediatr Radiol. (1996) 26:439–49. 10.1007/BF01377198 8662059

[11] Johnson TJ Jamous FG Kooistra A Zawada ET Iatrogenic chylothorax due to pleural cavity extravasation of total parenteral nutrition in two adults receiving nutrition through a peripherallyinserted catheter. Hosp Pract. (2010) 38:50–2. 10.3810/hp.2010.02.278 20469624

[12] Kua KL Whitehurst RM Jr Alrifai W Standley TB Zayek MM. Pleural effusion as a complication of a remotely placed catheter in a preterm infant. J Perinatol. (2013) 33:982–4. 10.1038/jp.2013.76 24276175

[13] Yu XH Yue SJ Wang MJ Cao CD Liao ZC Ding Y. Risk factors related to peripherally inserted central venous catheter nonselective removal in neonates. Biomed Res Int. (2018) 2018:3769376. 10.1155/2018/3769376 30003096 PMC5998161

[14] Jain A Deshpande P Shah P. Peripherally inserted central catheter tip position and risk of associated complications in neonates. J Perinatol. (2013) 33:307–12. 10.1038/jp.2012.112 22955288

[15] Alhatem A Estrella Y Jones A Algarrahi K Fofah O Heller DS. Percutaneous route of life: chylothorax or total parenteral nutrition-related bilateral pleural effusion in a neonate? Fetal and Pediatr Pathol. (2020). 10.1080/15513815.2020.1716897. [Epub ahead of print]. 32000556

[16] Baldinelli F Capozzoli G Pedrazzoli R Marzano N. Evaluation of the correct position of peripherally inserted central catheters: anatomical landmark vs. electrocardiographic technique. J Vasc Access. (2015) 16:394–8. 10.5301/jva.5000431 26109544

[17] Gao Y Liu Y Zhang H Fang F Song L. The safety and accuracy of ECG-guided ECC tip position verification applied in patients with atrial fibrillation. Ther Clin Risk Manag. (2018) 14:1075–81. 10.2147/TCRM.S156468 29922068 PMC5995413

[18] Li AM Jiao JG Zhang Y Tian L Miao JH Hao XL. A randomized controlled study of bedside electrocardiograph-guided tip location technique &the traditional chest radiography tip location technique for peripherally inserted central venous catheter in cancer patients. Indian J Med Res. (2018) 147:477–83. 10.4103/ijmr.IJMR_1120_16 30082572 PMC6094514

[19] Li WF Xu RC Fan D. Clinical application of electrocardiogram-guided tip positioning in peripheral inserted central catheters placement. J Cancer Res Ther. (2018) 14:887–91. 10.4103/jcrt.JCRT_46_18 29970671

[20] Ling QY Chen H Tang M Qu Y Tang BZ. Accuracy and safety study of intracavitary electrocardiographic guidance for peripherally inserted central catheter placement in neonates. J Perinat Neonatal Nurs. (2019) 33:89–95. 10.1097/JPN.0000000000000389 30676468

[21] Oliver G Jones M. ECG-based ECC tip verification system: an evaluation 5 years on. Br J Nurs. (2016) 25:S4–10. 10.12968/bjon.2016.25.19.S4 27792447

[22] Rosche N Stehr W. Evaluation of a magnetic tracking and electrocardiogram-based tip confirmation system for peripherally inserted central catheters in pediatric patients. J Infus Nurs. (2018) 41:301–8. 10.1097/NAN.0000000000000293 30188452

[23] Rossetti F Pittiruti M Lamperti M Graziano U Celentano D Capozzoli G. The intracavitary ECG method for positioning the tip of central venous access devices in pediatric patients: results of an Italian multicenter study. J Vasc Access. (2015) 16:137–43. 10.5301/jva.5000281 25198817

[24] Wang GR Guo L Jiang B Huang M Zhang J Qin Y. Factors influencing intracavitary electrocardiographic p-wave changes during central venous catheter placement. PLoS ONE. (2015) 10:e0124846. 10.1371/journal.pone.0124846 25915758 PMC4411117

[25] Yuan L Li RM Meng AF Feng YL Wu XC Yang YQ. Superior success rate of intracavitary electrocardiogram guidance for peripherally inserted central catheter placement in patients with cancer: a randomized open-label controlled multicenter study. PLoS ONE. (2017) 12:e0171630. 10.1371/journal.pone.0171630 28278167 PMC5344315

[26] Zhou LJ Xu HZ Liang JF Xu MF Yu J. Effectiveness of intracavitary electrocardiogram guidance in peripherally inserted central catheter tip placement in neonates. J Perinat Neonatal Nurs. (2017) 31:326–31. 10.1097/JPN.0000000000000264 28520655

[27] Liu G Hou WB Zhou C Yin YX LU ST Duan CH. Meta-analysis of intracavitary electrocardiogram guidance for peripherally inserted central catheter placement. J Vasc Access. (2019) 20:577–82. 10.1177/1129729819826028 30838913

[28] Yin YX Gao W Li XY Lu W Deng QH Zhao CY. Insertion of peripherally inserted central catheters with intracavitary electrocardiogram guidance: a randomized multicenter study in China. J Vasc Access. (2019) 20:524–9. 10.1177/1129729818819732 30596472 PMC6699060

[29] Weber MD Himebauch AS Conlon T. Repositioning of malpositioned peripherally inserted central catheter lines with the use of intracavitary electrocardiogram: a pediatric case series. J Vasc Access. (2019) 21:259–64. 10.1177/1129729819865812 31364466

[30] Katheria AC Fleming SE Kim JH. A randomized controlled trial of ultrasound-guided peripherally inserted central catheters compared with standard radiograph in neonates. J Perinatol. (2013) 33:791–4. 10.1038/jp.2013.58 23765173

[31] Telang N Sharma D Pratap OT Kandraju H Murki S. Use of real-time ultrasound for locating tip position in neonates undergoing peripherally inserted central catheter insertion: a pilot study. Indian J Med Res. (2017) 145:373–6. 10.4103/ijmr.IJMR_1542_14 28749401 PMC5555067

[32] Srinivasan HB Tjin-A-Tam A Galang R Hecht A Srinivasan G. Migration patterns of peripherally inserted central venous catheters at 24hours postinsertion in neonates. Am J Perinatol. (2013) 30:871–4. 10.1055/s-0033-1333672 23381907

